# A Novel Kv7.3 Variant in the Voltage-Sensing S_4_ Segment in a Family With Benign Neonatal Epilepsy: Functional Characterization and *in vitro* Rescue by β-Hydroxybutyrate

**DOI:** 10.3389/fphys.2020.01040

**Published:** 2020-09-04

**Authors:** Francesco Miceli, Lidia Carotenuto, Vincenzo Barrese, Maria Virginia Soldovieri, Erin L. Heinzen, Arthur M. Mandel, Natalie Lippa, Louise Bier, David B. Goldstein, Edward C. Cooper, Maria Roberta Cilio, Maurizio Taglialatela, Tristan T. Sands

**Affiliations:** ^1^Department of Neuroscience, University of Naples “Federico II”, Naples, Italy; ^2^Department of Medicine and Health Science, University of Molise, Campobasso, Italy; ^3^Eshelman School of Pharmacy, Division of Pharmacotherapy and Experimental Therapeutics, University of North Carolina at Chapel Hill, Chapel Hill, NC, United States; ^4^Institute for Genomic Medicine, Columbia University Irving Medical Center, New York, NY, United States; ^5^Department of Neurology, Columbia University Irving Medical Center, New York, NY, United States; ^6^Departments of Neurology, Neuroscience, and Molecular and Human Genetics, Baylor College of Medicine, Houston, TX, United States; ^7^Department of Pediatrics and Institute of Experimental and Clinical Research, Cliniques universitaires Saint-Luc, Université catholique de Louvain, Brussels, Belgium

**Keywords:** *KCNQ*, BFNE, encephalopathy, channelopathies, ketogenic diet

## Abstract

Pathogenic variants in *KCNQ2* and *KCNQ3*, paralogous genes encoding Kv7.2 and Kv7.3 voltage-gated K^+^ channel subunits, are responsible for early−onset developmental/epileptic disorders characterized by heterogeneous clinical phenotypes ranging from benign familial neonatal epilepsy (BFNE) to early−onset developmental and epileptic encephalopathy (DEE). *KCNQ2* variants account for the majority of pedigrees with BFNE and *KCNQ3* variants are responsible for a much smaller subgroup, but the reasons for this imbalance remain unclear. Analysis of additional pedigrees is needed to further clarify the nature of this genetic heterogeneity and to improve prediction of pathogenicity for novel variants. We identified a BFNE family with two siblings and a parent affected. Exome sequencing on samples from both parents and siblings revealed a novel *KCNQ3* variant (c.719T>G; p.M240R), segregating in the three affected individuals. The M240 residue is conserved among human Kv7.2-5 and lies between the two arginines (R5 and R6) closest to the intracellular side of the voltage-sensing S_4_ transmembrane segment. Whole cell patch-clamp recordings in Chinese hamster ovary (CHO) cells revealed that homomeric Kv7.3 M240R channels were not functional, whereas heteromeric channels incorporating Kv7.3 M240R mutant subunits with Kv7.2 and Kv7.3 displayed a depolarizing shift of about 10 mV in activation gating. Molecular modeling results suggested that the M240R substitution preferentially stabilized the resting state and possibly destabilized the activated state of the Kv7.3 subunits, a result consistent with functional data. Exposure to β-hydroxybutyrate (BHB), a ketone body generated during the ketogenic diet (KD), reversed channel dysfunction induced by the M240R variant. In conclusion, we describe the first missense loss-of-function (LoF) pathogenic variant within the S_4_ segment of Kv7.3 identified in patients with BFNE. Studied under conditions mimicking heterozygosity, the M240R variant mainly affects the voltage sensitivity, in contrast to previously analyzed BFNE Kv7.3 variants that reduce current density. Our pharmacological results provide a rationale for the use of KD in patients carrying LoF variants in Kv7.2 or Kv7.3 subunits.

## Introduction

Voltage-gated potassium (K^+^) channels (Kv channels) regulate the resting membrane potential and set the threshold and duration of the action potential in excitable cells. Among these, Kv7.2 and Kv7.3 voltage-gated K^+^ subunits, encoded by the *KCNQ2* and *KCNQ3* genes, are expressed in the central and peripheral nervous system (Brown and Adams, 1980; Wang et al., 1998). These subunits form homo- and heterotetrameric channels underlying the M-current (*I*_KM_), a non-inactivating K^+^ current with slow activation and deactivation kinetics that activates at the threshold potential of about −60/−50 mV, thus regulating the resting membrane potential and suppressing repetitive neuronal firing (Brown and Adams, 1980).

Pathogenic variants in *KCNQ2* cause early-onset epilepsies with wide phenotypic heterogeneity (Allen et al., 2014; Miceli et al., 2018). Indeed, some variants have been identified in patients with benign familial neonatal epilepsy (BFNE), an autosomal-dominant epilepsy with seizures affecting otherwise healthy infants in the first days of life and spontaneously disappearing over the next several months, with mostly normal neurocognitive development (Jentsch, 2000; Singh et al., 2003; Soldovieri et al., 2006). At the severe end of the *KCNQ2* spectrum is an early-onset developmental and epileptic encephalopathy (DEE) characterized by recurrent seizures starting in the neonatal period and neurodevelopmental disability (Weckhuysen et al., 2012). While more than 300 pathogenic variants have been described in *KCNQ2*, few variants in *KCNQ3* have been detected, mostly in families with BFNE. In addition, *de novo* variants in *KCNQ3* have been rarely described in children with DEE (Allen et al., 2013; Grozeva et al., 2015; Miceli et al., 2015; Ambrosino et al., 2018; Lauritano et al., 2019), intellectual disability (ID) apparently without epilepsy (Rauch et al., 2012; Deciphering Developmental Disorders, 2017), cortical visual impairment (Bosch et al., 2016), and in patients with ID and autism (Sands et al., 2019).

In most children affected with *KCNQ2*- or *KCNQ3*-related BFNE, seizures are controlled with conventional antiepileptic drugs, including sodium channel blockers (Sands et al., 2016). Instead, few options are available for patients with the most severe forms of *KCNQ2*- or *KCNQ3*-related disorders; in addition to sodium-channel blockers such as carbamazepine and phenytoin which appear to be highly effective (Pisano et al., 2015), ezogabine, a selective Kv7 channel activator, has been shown to improve seizure control and development in patients with Kv7.2 loss-of-function (LoF) variants (Millichap et al., 2016). Unfortunately, because of its unfavorable risk/benefit ratio, ezogabine has been withdrawn from the market. Among non-pharmacological therapies, ketogenic diet (KD) has been recently shown to be particularly effective in children with DEE caused by *KCNQ2* variants (Ko et al., 2018), but the mechanisms of action are not completely understood and there are no data on the effects on *KCNQ3* related disorders. KD is a low carbohydrate, high-fat, adequate-protein diet regimen that shifts the primary fuel source for neuronal activity from glucose to endogenous ketones: acetone, acetoacetate, and β-hydroxybutyrate (BHB). KD likely improves seizure control through a variety of mechanisms such as inhibition of the glycolysis, disruption of glutamatergic synaptic transmission, and activation of ATP-sensitive potassium channels (Lutas and Yellen, 2013). Recently, BHB has been shown to directly and specifically activate Kv7 channels containing Kv7.3 subunits by increasing current sensitivity to voltage (Manville et al., 2018, 2020).

In the present manuscript, we report the clinical, molecular and functional properties of a BFNE family carrying a novel *KCNQ3* variant (c.719T>G; p.M240R) segregating with epilepsy in the three affected individuals. Whole cell patch-clamp recordings in Chinese hamster ovary (CHO) cells revealed that homomeric Kv7.3 M240R channels were not functional, whereas heteromeric channels incorporating Kv7.3 M240R mutant subunits with Kv7.2 and Kv7.3 displayed a depolarizing shift of about 10 mV in activation gating, consistent with a LoF *in vitro* effect. Consistent with these functional data, molecular modeling suggested that the M240R substitution preferentially stabilized the resting state and possibly destabilize the activated state of the Kv7.3 subunits. Finally, we demonstrate that BHB was able to reverse the functional alterations observed in heteromeric channels carrying Kv7.3 M240R subunits.

## Materials and Methods

### Patients

The BFNE family was referred for an epilepsy genetics research study by their treating clinician and written informed consent was obtained. Exome sequencing was performed in both parents and each sibling, and the presence of c.719T > G (p.M240R) in affected members was confirmed by Sanger sequencing in a clinical genetics laboratory. The study was approved by the human research ethics committee of Columbia University Irving Medical Center.

### Mutagenesis and Heterologous Expression of *KCNQ2* and *KCNQ3* cDNAs

Mutations were engineered in *KCNQ3* human cDNA cloned into pcDNA3.1 by QuikChange site-directed mutagenesis (Agilent Technologies Italia SpA, Milan, Italy), as previously described (Miceli et al., 2013). Channel subunits were expressed in CHO cells by transient transfection. CHO cells were grown in 100 mm plastic Petri dishes in Dulbecco’s modified Eagle’s medium (DMEM) containing 10% foetal bovine serum (FBS), L-glutamine (0.1 mM), penicillin (50 U/ml), and streptomycin (50 μg/ml) in a humidified atmosphere at 37°C with 5% CO_2_. For electrophysiological experiments, cells were seeded on glass coverslips (Carolina Biological Supply Company, Burlington, NC, United States) and transfected on the next day with the appropriate cDNAs using Lipofectamine 2000 (Invitrogen, Milan, Italy) according to the manufacturer’s protocol. A plasmid encoding for enhanced green fluorescent protein (Clontech laboratories, Inc., Palo Alto, CA, United States) was used as transfection marker; total cDNA in the transfection mixture was kept constant at 4 μg.

### Whole-Cell Electrophysiology

Currents from CHO cells were recorded at room temperature (20–22°C) 1–2 days after transfection, using a commercially available amplifier (Axopatch 200A, Molecular Devices, Union City, CA, United States) and the *whole-cell* configuration of the patch-clamp technique, with glass micropipettes of 3–5 MΩ resistance. The extracellular solution contained (in millimolar): 138 NaCl, 2 CaCl_2_, 5.4 KCl, 1 MgCl_2_, 10 glucose, and 10 HEPES, pH 7.4 with NaOH. The pipette (intracellular) solution contained (in millimolar): 140 KCl, 2 MgCl_2_, 10 EGTA, 10 HEPES, 5 Mg-ATP, pH 7.3–7.4 with KOH. The pCLAMP software (version 10.0.2) was used for data acquisition and analysis. Current densities (expressed in picoamperes per picofarad) were calculated as peak K^+^ currents divided by *C*. Data were acquired at 0.5–2 kHz and filtered at 1–5 kHz with the four-pole lowpass Bessel filter of the amplifier. No corrections were made for liquid junction potentials. To generate conductance-voltage curves, the cells were held at −80 mV, then depolarized for 1.5 s from −80 mV to +20/+80 mV in 10 mV increments, followed by an isopotential pulse at 0 mV of 300 ms duration; the current values recorded at the beginning of the 0 mV pulse were measured, normalized, and expressed as a function of the preceding voltages. Data were fit to a Boltzmann distribution of the following form *y* = max/[1 + exp(*V*_1__/__2_−*V*)/*k*], where *V* is the test potential, *V*_1__/__2_ the half-activation potential, and *k* the slope factor.

For current-activation kinetics analysis, the current traces recorded in response to incremental voltage steps were fitted to a double-exponential function and then a single time constant, representing the weighted average of the slow and fast components, was obtained by using the following equation: τ = (τ_f_ *A*_f_ + τ_s_ *A*_s_)/(*A*_f_ + *A*_s_), where *A*_f_ and *A*_s_ indicate the amplitude of the fast and slow exponential components, τ_f_ and τ_s_ the time constants of each component (Miceli et al., 2013).

### Structural Modeling

Three-dimensional models of K_v_7.3 subunits in different gating states were generated by using as templates the coordinates of resting and activated states of the K_v_1.2/2.1 chimera (Long et al., 2007; PDB accession number 2R9R; 26% of sequence identity with K_v_7.3) subjected to long (>200 μs) molecular dynamic simulations (Jensen et al., 2012). Modeling of the S_1_-S_4_ VSD in each state was performed with SWISS-MODEL, as described (Sands et al., 2019). The models were optimized through all-atom energy minimization by using the GROMOS96 implementation of Swiss-PDBViewer, and analyzed using both the DeepView module of Swiss-PDBViewer (version 4.0.1^1^) and PyMOL^2^.

### Statistics

Data are expressed as the mean ± SEM. Statistically significant differences between the data were evaluated with the Student’s *t*-test (*p* < 0.05).

## Results

### Clinical and Genetic Features of the BFNE Pedigree With a Novel *KCNQ3* Variant

#### Case II-1

A term male neonate was hospitalized for seizures starting in the first week of life. The seizures lasted less than 1 min, recurring every 2 h. Electroencephalography (EEG) recorded variable lateralization of ictal onset. Clinically, there was bilateral arm stiffening, rightward head version, and right leg stiffening, lasting 45 s. Workup for acute etiologies, including an MRI of the brain, was unrevealing. The child was treated with phenobarbital, but continued to have seizures after hospital discharge. He was cross-titrated onto levetiracetam from 3 weeks of age and seizures stopped around that time. Levetiracetam was weaned at 12 months. At 18 months he was diagnosed with isolated language delay (one standard deviation below expected) and started speech therapy. There was no concern for autism.

#### Case II-2

Nineteen months later, a female sibling was born. She began having episodes on the 5th day of life, characterized by limb stiffening and eye rolling. EEG confirmed seizures. Workup included a normal MRI of the brain. She was treated with phenobarbital and levetiracetam, which were weaned off at 4 and 6 months, respectively. She had recurrence with two back-to-back seizures at 22 months, and she was started on oxcarbazepine with seizure freedom. Follow-up EEGs have been normal. Developmental milestones have all been met on time as of 26 months.

**FIGURE 1:**
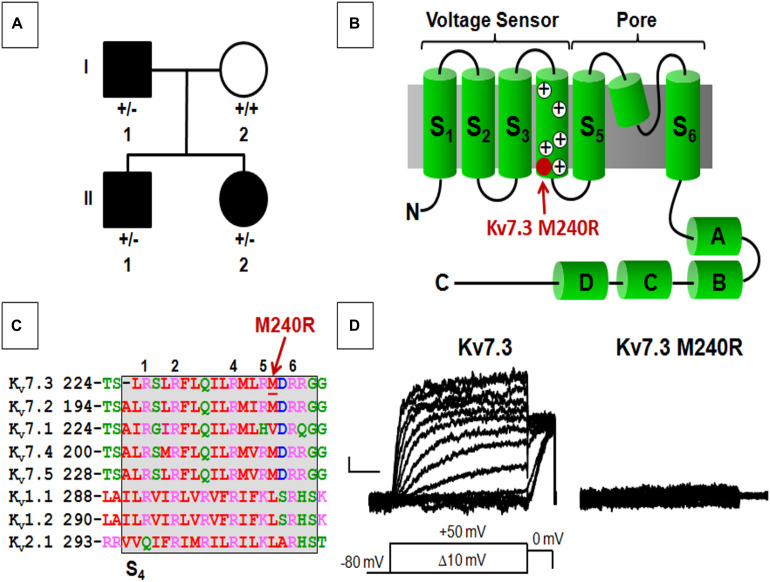
Pedigree, schematic representation of a single Kv7.3 subunit, and functional analysis of mutation at position 240. **(A)** Pedigree of the family. **(B)** Topological representation of a single Kv7 subunit. Arrows indicate the location of the mutation investigated (shaded in red). **(A–D)** Indicate the four putative alpha-helical domains identified in the Kv7 C-terminal region. **(C)** Sequence alignment of the S_4_ segments of the indicated Kv subunits (www.ebi.ac.uk/Tools/psa/). Residues are colored according to the following scheme: magenta represents basic; blue represents acid; red represents non-polar; green represents polar. 1, 2, 4, 5, and 6 refer to the positively charged arginines numbered according to their position along the S_4_ primary sequence. **(D)** Macroscopic currents from the indicated homomeric channels. Current scale, 50 pA; time scale, 0.2 s.

Family history was notable for an isolated seizure in the father (case I-1) during infancy. The father’s interictal EEG was normal, the seizure did not recur, and he was not treated with anti-seizure medication. No seizure was ever described in the mother (case I-2; Figure 1A).

Exome sequencing, performed in all four family members, demonstrated a novel missense variant in *KCNQ3*, c.719T > G (p.M240R), in the two siblings and the affected father (Figure 1A), thus segregating with the phenotype. The variant is absent from gnomAD and predicted to be deleterious (polyphen-2, 0.989; SIFT, 0.9122; CADD score 27.3). The M240 residue lies between the R5 (R239) and R6 (R242) positions of the voltage-sensing S_4_ transmembrane segment within the voltage-sensing domain (VSD; Figure 1B). This non-polar residue is conserved among human Kv7.2-5 subunits, but not in Kv7.1 and other Kv channels such as Kv1.1, Kv1.2, and Kv2.1, although residues with similar physicochemical properties are present at this position (Figure 1C).

### Functional and Pharmacological Properties of Homomeric and Heteromeric Channels Carrying Kv7.3 M240R Subunits

Heterologous expression of wild-type Kv7.3 subunits led to the appearance of voltage-dependent K^+^ −selective currents characterized by a rather slow time-course of activation and deactivation and a threshold for current activation of around −50 mV; the macroscopic K^+^ currents density at + 20 mV was 12.6 ± 1.1 pA/pF, similarly to previously reported values (Wang et al., 1998; Sands et al., 2019). By contrast, no measurable currents were recorded in cells expressing Kv7.3 M240R subunits, consistent with the variant causing a complete LoF effect (Figure 1D and Table 1).

**TABLE 1:** Biophysical and pharmacological properties of mutant Kv7.3 channels.

	*n*	*V*_1/2_ (mV)	*k* (mV/e-fold)	Current density at + 20 mV (pA/pF)	Blockade by 3 mM TEA (%)	100 μM BHB (Δ V, mV)
CHO	10	–	–	0.5 ± 0.05	–	–
Kv7.3	26	−37.2 ± 1.1*	7.2 ± 0.4*	12.6 ± 1.1*	8.0 ± 2.1*	–
Kv7.3 M240R	8	–	–	0.5 ± 0.2	–	–
Kv7.2	11	−26.8 ± 1.3	13.2 ± 0.9	42.1 ± 2.5	85.1 ± 3.1*	–
Kv7.2 + Kv7.3	18	−27.7 ± 1.4	11.2 ± 0.5	126.4 ± 17.9	50.1 ± 4.3	−8.1 ± 1.1
Kv7.2 + Kv7.3 M240R	7	18.1 ± 4.9*	22.6 ± 0.8*	70.2 ± 10.4*	45.2 ± 5.1	–
Kv7.2 + Kv7.3 + Kv7.3 M240R	18	−15.6 ± 1.7*	15.5 ± 0.7*	115.0 ± 15.2	48.5 ± 5.2	−8.7 ± 1.1

*TEA, tetraethylammonium; CHO, Chinese hamster ovary. ^∗^*p* < 0.05 vs. Kv7.2 + Kv7.3.*

**FIGURE 2:**
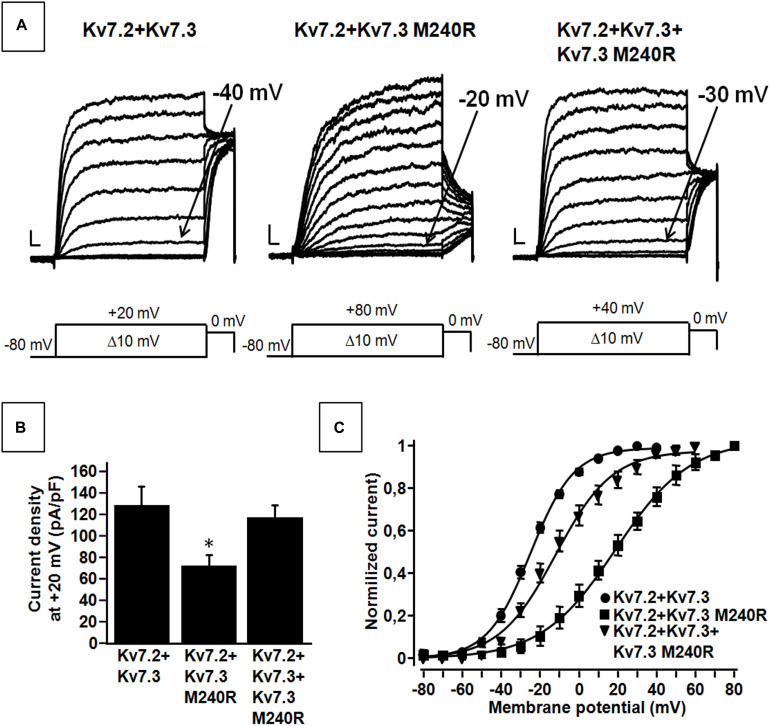
Functional properties of heteromeric channels incorporating subunits carrying the benign familial neonatal epilepsy (BFNE) variant. **(A)** Macroscopic current traces from the indicated heteromeric channels in response to the indicated voltage protocol. Current scale, 200 pA; time scale, 0.1 s. **(B)** Current density from the indicated heteromeric channels calculated at +20 mV. The asterisk indicates a statistically-significant value versus Kv7.2 + Kv7.3. **(C)** Conductance/voltage curves for Kv7.2 + Kv7.3, Kv7.2 + Kv7.3 M240R, Kv7.2 + Kv7.3 + Kv7.3M240R. Continuous lines indicate Boltzmann fits of the experimental data. Current scale, 200 pA; time scale, 0.1 s. Each data point is the mean (SEM) of 7–18 cells recorded in at least three separate experimental sessions.

Kv7.3 subunits assemble with Kv7.2 subunits to form *I*_KM_ (Wang et al., 1998). Co-expression of Kv7.2 and Kv7.3 subunits generated currents which were considerably larger than those recorded upon expression as Kv7.2 or Kv7.3 subunits alone; in addition, currents from Kv7.2 + Kv7.3 heteromeric channels displayed an increased sensitivity to blockade by tetraethylammonium (TEA) when compared to homomeric Kv7.3 channels (Table 1). When compared to currents from wild-type Kv7.2 + Kv7.3 subunits, co-expression of Kv7.3 M240R with Kv7.2 subunits caused a marked rightward shift (by about + 40 mV) of the activation gating, a significant decrease of slope of the G/V curve (see section Materials and Methods for details), a reduced current density by about 50% at depolarized potential, with no change in sensitivity to TEA blockade (Figures 2A–C and Table 1). The marked rightward shift of the G/V curves of the heteromeric Kv7.2 + Kv7.3 M240R channels markedly reduced current density at the physiologically relevant potentials of −50/−40 mV (Figure 2C). The activation kinetics of Kv7.2 + Kv7.3-M240R currents were slower than those of Kv7.2 + Kv7.3, particularly at more depolarized potentials. In fact, τ_activation_ at + 20 mV were 142 ± 19 ms and 274 ± 71 ms, for Kv7.2 + Kv7.3 and Kv7.2 + Kv7.3-M240R, respectively. In order to assess the influence of the depolarizing pulse length on the current steady-state properties from Kv7.2 + Kv7.3-M240R-expressing channels, additional experiments in which the pulse length was increased from 1.5 to 3 s were performed. The results obtained revealed no significant difference in the *V*_1/2_ and *k* values on Kv7.2 + Kv7.3-M240R current using the two protocols; indeed, the *V*_1/2_ and the *k* values were, respectively, + 12.2 ± 7.6 and + 10.2 ± 5.4 mV (*n* = 4; *p* > 0.05), and 23.0 ± 3.6 and 23.5 ± 1.4 mV/e-fold (*n* = 4; *p* > 0.05) for Kv7.2 + Kv7.3- and Kv7.2 + Kv7.3-M240R-expressing cells.

Altogether these data suggest that Kv7.3 M240R subunits are able to form heteromeric channels with Kv7.2 subunits, although their currents display gating properties very different from Kv7.2 + Kv7.3 heteromeric channels. To replicate *in vitro* the genetic combination occurring in the affected family members who are heterozygous for the pathogenic allele (Individuals I-1, II-1, and II-2), functional studies were also carried out upon transfection of cDNAs for Kv7.2 + Kv7.3 + Kv7.3 M240R at a cDNA ratio of 1:0.5:0.5. The current density measured in CHO cells expressing heteromeric channels formed by the described subunit combinations was very similar to that recorded in cells expressing Kv7.2 + Kv7.3 subunits at a transfection ratio 1:1 (mimicking a healthy individual) (Figures 2A,B and Table 1) and no difference in the activation kinetics was observed between the Kv7.2 + Kv7.3 and Kv7.2 + Kv7.3 + Kv7.3 M240R currents; indeed the τ_activation_ at + 20 mV were 142 ± 19 ms and 128 ± 28 ms, respectively. By contrast, the midpoint of activation of the currents recorded upon Kv7.2 + Kv7.3 + Kv7.3 M240R subunit co-expression was right-shifted by about 10 mV when compared to Kv7.2 + Kv7.3 (Figures 2A–C and Table 1), thus confirming LoF effects of the mutant subunits also when incorporated into channel tetramers with Kv7.2 + Kv7.3 subunits.

### Structural Basis for the LoF Effect by the Kv7.3 M240R Substitution

**FIGURE 3:**
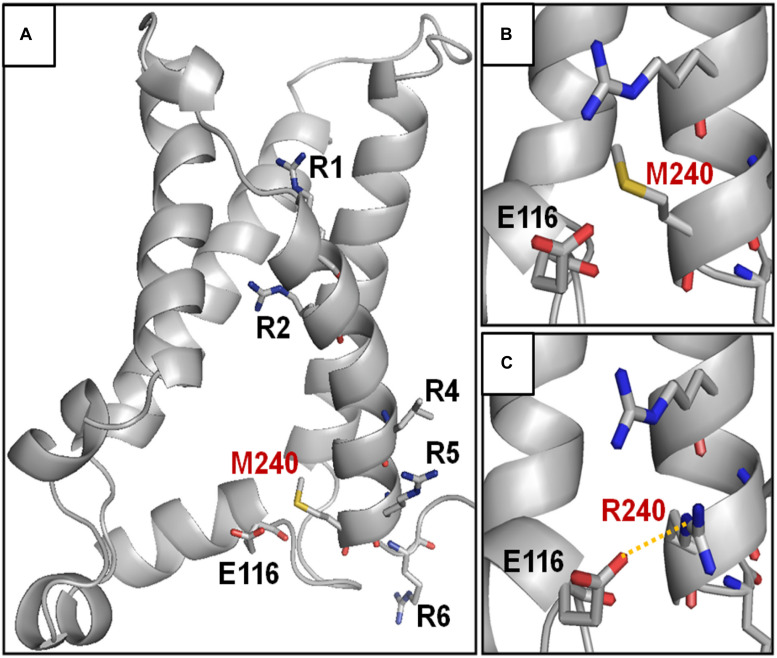
Structural modeling of homomeric Kv7.3 channel subunits in resting gating states. **(A)** Homology model of a homotetrameric Kv7.3 channel subunits, obtained as described in the section “Materials and Methods.” **(B,C)** Enlarged views showing the N-terminal domain encompassing the E116 residue in the wild-type **(B)** or M240R mutant **(C)** subunits, showing the occurrence the E116-R240 polar interaction (highlighted in yellow).

The herein identified M240R variant introduces an extra positively charged residue into the S_4_ segment of Kv7.3 subunits, between R5 and R6. To achieve a better understanding of the possible role of the M240 residue in the gating process and of the structural consequences of its replacement with an R, we built homology models of a Kv7.3 subunit in both resting and activated states, as previously described (Jensen et al., 2012; Sands et al., 2019). Our structural models suggested that, no electrostatic interaction between the M240 residue side chain and surrounding protein residues occurs in both the resting and the activated VSD configurations (Figure 3A); in particular, in the resting VSD state and in two of the four subunits, the M240 side chain points toward the N-terminal region of the same subunits, whereas in the other two subunits it points in the opposite direction, namely toward the S_5_ segment (Figure 3B). Substitution of the non-polar M residue at position 240 with an R introduces a novel electrostatic interaction between R240 side chain and a highly conserved negatively charged N-terminal glutamate (E116) in one Kv7.3 subunit. This interaction only occurred in the resting state of the VSD (Long et al., 2007) (Figure 3C). Instead, VSD movement during the activation process translated the R240 side chain in a tight pocket surrounded by non-polar residues, possibly destabilizing the tight network of hydrophobic interactions within this pocket. As a result, our model suggests that the M240R substitution may preferentially stabilize the closed state and possibly destabilize the activated state of the Kv7.3 subunits.

### Pharmacological Effects of BHB Exposure on Heteromeric Channels Incorporating the Kv7.3 Epilepsy-Causing Variant

**FIGURE 4:**
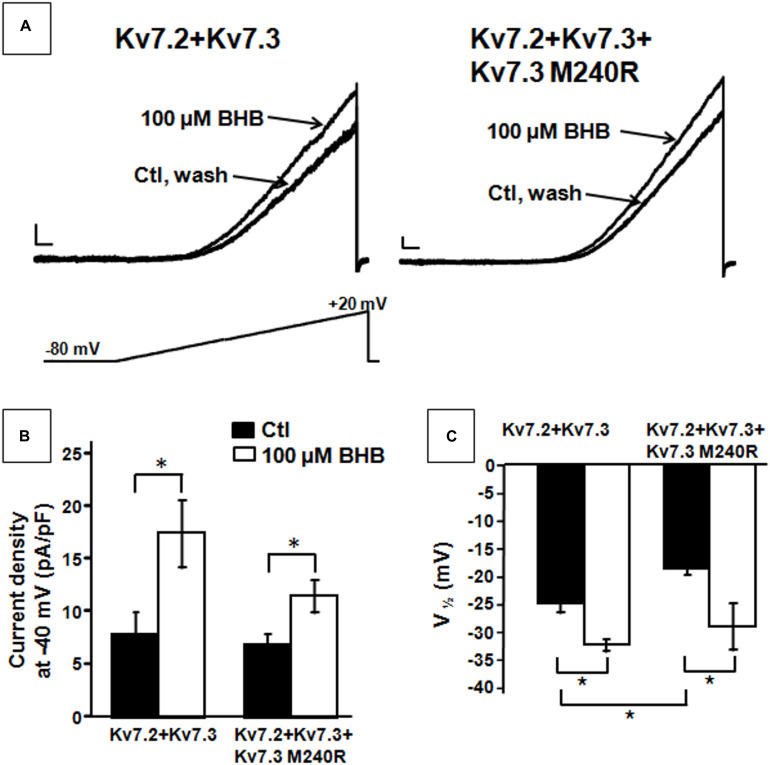
Effect of β-hydroxybutyrate (BHB) on heteromeric Kv7.2 + Kv7.3, Kv7.2 + Kv7.3 + Kv7.3 M240R channels. **(A)** Current responses from the indicated heteromeric channels to voltage ramps from −80 to +20 mV. **(B,C)** Quantification of the effects of 100 μM BHB on the indicated heteromeric channels. Data are expressed as current density calculated at −40 mV **(B)** and in the *V*_1/2_ values. Current scale, 200 pA; time scale, 200 ms. * indicates *p* < 0.05 vs. corresponding controls.

The results described suggest that heteromeric channels incorporating Kv7.3 M240R subunits display a reduced sensitivity to voltage, strongly suggesting a LoF pathogenic mechanism. Given that BHB has been recently shown to potentiate Kv7.3 and Kv7.2 + Kv7.3 currents by a mechanism opposite to that introduced by the M240R variant (Manville et al., 2018, 2020), we further evaluated its ability to counteract *in vitro* the described functional alteration. We tested the effects of the BHB using a voltage protocol in which Kv7.2 + Kv7.3 and Kv7.2 + Kv7.3 + Kv7.3 M240R currents were activated by 3 s voltage ramps from −80 to +20 mV. Perfusion with 100 μM BHB enhanced ramp-evoked Kv7.2 + Kv7.3 and Kv7.2 + Kv7.3 + Kv7.3 M240R currents; this effect was reversible since the currents returned to basal values after about 10 s upon BHB removal from the bath (Figure 4A). In addition, steady-state experiments revealed that at −40 mV, a membrane potential value close to the activation threshold, 100 μM BHB increased Kv7.2 + Kv7.3 and Kv7.2 + Kv7.3 + Kv7.3 M240R currents (Figure 4B) and also caused a 10 mV negative shift in the G/V curve (Figure 4C). Interestingly, the *V*_1/2_ value of Kv7.2 + Kv7.3 + Kv7.3 M240R currents upon BHB exposure was similar to that of Kv7.2 + Kv7.3-expressing cells (Figure 4C and Table 1), suggesting the ability of the BHB to restore Kv7.2 + Kv7.3 + Kv7.3 M240R currents to that of wild-type.

## Discussion

Inherited variants in *KCNQ2* or *KCNQ3* cause BFNE, a neonatal-onset familial epilepsy, characterized by recurrent focal tonic seizures in otherwise well infants (Sands et al., 2016). While seizure onset is most often in the first days of life, seizures can present later in infancy (Zara et al., 2013), as demonstrated by the father in our pedigree, or not at all, as penetrance is incomplete. Seizures tend to remit over the first year, but ∼30% of individuals have seizures later in life (Grinton et al., 2015), as illustrated by case II-2.

Most BFNE families carry pathogenic variants in *KCNQ2*, whereas only a small percentage carry *KCNQ3* variants (Grinton et al., 2015; Sands et al., 2016; Miceli et al., 2017, 2018). *KCNQ2* variants responsible for BFNE are missense, stop-gain, frameshift, splice variants, and deletions randomly distributed along the entire primary sequence of the subunit. In contrast, BFNE-causing *KCNQ3* variants are all missense, affecting specific residues located in the pore region of the channel (S_5_–S_6_ and intervening loop; Table 2). Prior studies have reported 13 such variants, each affecting a different Kv7.3 residue located in and around the pore (Table 2); in addition, four Kv7.3 variants affecting residues in the long C-terminus have also been described, although the pathogenic role of these variants appears questionable due to their ample representation in the gnomAD population database without the benefit of supportive functional data (N821S, Bassi et al., 2005; R780C, Zara et al., 2013), or with functional data that fail to support a disease association (N468S, Singh et al., 2003; P574S, Miceli et al., 2009). Out of the 13 *KCNQ3* variants causing BFNE, those nine with supportive functional data lie within a span of 51 residues (V279 to R330) in this region. Functional analyses of these variants to date, e.g., V279F, I317T, R330C, and R330L, have mostly demonstrated a reduction in maximal current with no effect on the voltage-dependence of activation (Soldovieri et al., 2014; Miceli et al., 2015; Maljevic et al., 2016), consistent with these variants being in the pore. Only W309R induced both a reduction in currents (by about 60%) and a rather small (3–4 mV) rightward shift in the *V*_1/2_ value, when expressed together with Kv7.2 and Kv7.3 (Uehara et al., 2008).

**TABLE 2:** Missense variants reported for *KCNQ3*.

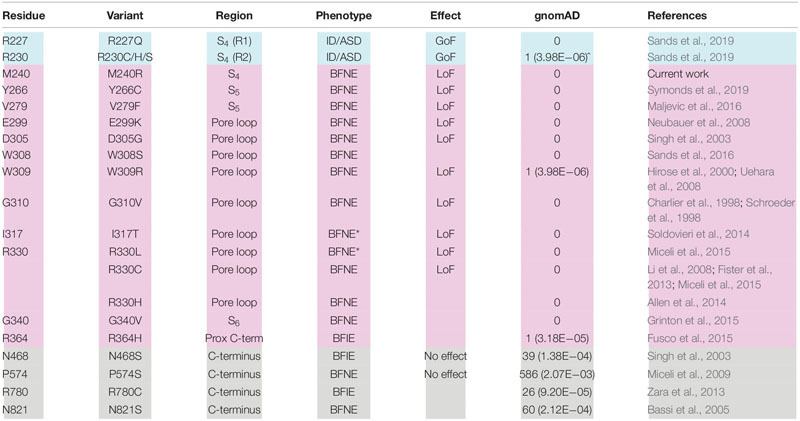

Table gives number of observations of each variant (frequency) in gnomAD, associated phenotype, region within Kv7.3, and functional consequences *in vitro* if known; blue, intellectual disability (ID) with or without autism spectrum disorder (ASD); purple, benign familial neonatal epilepsy (BFNE) or benign infantile epilepsy (BFIE) phenotypes; gray, variants of questionable pathogenicity; LoF, loss-of-function; GoF, gain-of-function; * individuals with mild/moderate ID reported; ^ mosaic individual.

In the present work, we report a BFNE family carrying the first variant in *KCNQ3* located in the S_4_ helix of the VSD (M240R). Functional studies revealed that the M240R variant abolishes channel function in homomeric configuration, but that this is partially rescued in heteromeric channels with wild-type Kv7.2 and/or Kv7.2/3 subunits. Differently from all other Kv7.3 BFNE variants characterized to date, M240R subunits, when expressed with Kv7.2 and Kv7.3 subunits, show a significant decrease of channel sensitivity to voltage of about 10 mV, without major changes in pore properties and heteromerization. These functional results suggest that the introduction of an additional positively charged residue at the bottom of S_4_ in Kv7.3 subunits destabilizes the activated conformation of the voltage sensor, thereby impeding pore opening. Structural modeling provides a potential explanation for such a conclusion; indeed, introduction of an R at position 240 stabilizes the VSD resting state by forming a novel strong ionic interaction with an highly conserved residue in the N-terminus; in addition, the larger R side chain may destabilize the tight network of hydrophobic interaction occurring in the activated state. Further studies using residue swapping or charge reversion are needed to confirm the potential interaction between the R240 and E116 residues. The available data do not allow us to determine whether the G/V shift introduced by the mutation originates from the participation of the 240 residue in voltage-sensing or in the subsequent steps along the activation pathway leading to pore opening. However, the fact that recent structural data from human Kv7.1 (Sun and MacKinnon, 2020), reveal that the V241 residue, corresponding to the M240 residue in Kv7.3, is located within a key region for VSD-pore electro-mechanical coupling (Hou et al., 2020), raises the possibility that this residue may play a similar functional role also in Kv7.3. Therefore, a LoF mechanism appears mainly responsible for BFNE pathogenesis in our family. Similar conclusions have been reached for BFNE-causing variants in Kv7.2, where haploinsufficiency, corresponding to an *I*_KM_ reduction of only ∼25%, appears responsible for disease pathogenesis (Jentsch, 2000).

Benign familial neonatal epilepsy with normal neurocognitive development is not the only phenotype associated with variants in Kv7.3. In fact, individuals with mild/moderate ID have been described in BFNE pedigrees (I317T, R330L; Soldovieri et al., 2014; Miceli et al., 2015); moreover, *de novo* variants in *KCNQ3* have been described in children with DEE (Allen et al., 2013; Grozeva et al., 2015; Miceli et al., 2015; Ambrosino et al., 2018; Lauritano et al., 2019), ID apparently without epilepsy (Rauch et al., 2012; Deciphering Developmental Disorders, 2017), cortical visual impairment (Bosch et al., 2016), and in patients with ID and autism (Sands et al., 2019). Such phenotypic heterogeneity is at least in part correlated with variant-specific functional effects, by yet unknown mechanisms; in fact, opposite to BFNE variants causing LoF, gain-of-function (GoF) variants cause non-verbal ID, autism, and prominent sleep-activated multifocal epileptiform EEG discharges without neonatal seizures (Singh et al., 2003; Sands et al., 2019).

The observation that epilepsy-associated variants in *KCNQ2* are more than 10 times more frequent than those in *KCNQ3*, suggests that *KCNQ3* tolerates variation better than *KCNQ2.* Several pieces of evidence support this view; as an example, while heterozygous frameshift variants in *KCNQ2* are frequent causes of BFNE (Miceli et al., 2018), no heterozygous pathogenic frameshift *KCNQ3* variant has ever been associated with a human phenotype. In addition, no individual carrying *KCNQ2* frameshift variants in homozygosity has ever been described, as minimal *KCNQ2* residual activity is probably essential for survival (Lauritano et al., 2019); by contrast, two recent studies reported the occurrence of homozygous frameshift variants in *KCNQ3* (each inherited from an asymptomatic parent) in patients with developmental delay and neonatal seizures (Kothur et al., 2018; Lauritano et al., 2019), demonstrating that, homozygous variants in *KCNQ3* are compatible with life. Moreover, a distinct functional role of these two genes emerging from developmental and genetic studies in humans, appears also to be recapitulated in mice. In fact, while *KCNQ2* homozygous KO mice died at birth, homozygous *KCNQ3* KO mice showed no seizures and survived until adulthood (Tzingounis and Nicoll, 2008; Kim et al., 2016); similarly, conditional deletion of *KCNQ2* in cortical pyramidal neurons increased neuronal excitability and decreased lifespan, whereas deletion of *KCNQ3* neither increased pyramidal neurons excitability nor affected mice survival (Soh et al., 2014).

Notably, in heteromeric channels, the functional effects of the M240R variant in Kv7.3 herein described in a BFNE family are quantitatively and qualitatively very similar to those triggered by the R213Q variant in Kv7.2, the latter identified in sporadic patients with DEE (Weckhuysen et al., 2012; Millichap et al., 2016). The fact that similar, dramatic *in vitro* functional consequences are associated to a severe phenotype in Kv7.2 and to self-limiting epilepsy in Kv7.3, further supports the hypothesis that Kv7.3 is more tolerant than Kv7.2 to genetic changes causing LoF effects. Among many others, such as inclusion/exclusion of both genes in panels for DEE, diagnostic NGS coverage, and epistatic compensation, an attractive explanation for such distinct functional role lies in the different developmental pattern of expression, with *KCNQ2* being expressed at earlier stages of development when compared to *KCNQ3*, in both mice and humans (Tinel et al., 1998; Kanaumi et al., 2008).

In patients with *KCNQ2*- and *KCNQ3*-related disorders, seizures are controlled with sodium channel blockers in most patients (Pisano et al., 2015; Sands et al., 2016). However, a percentage continues to have seizures, which may contribute to cognitive impairment. In addition, successful management of seizures does not address the neurodevelopmental disability that occurs in *KCNQ2* and *KCNQ3* DEEs. Efficacy of the KD, with greater than 90% seizure reduction, has been reported in patients with monogenic DEEs, including KCNQ2-DEE (Ko et al., 2018) and Dravet syndrome (Knupp and Wirrell, 2018). Our finding that the endogenous ketone BHB can activate heteromeric Kv7.2 + Kv7.3 channels containing Kv7.3 M240R subunits to the same extent as wild-type Kv7.2 + Kv7.3 channels (Manville et al., 2018, 2020), thereby counteracting the underlying pathophysiology, suggests that the KD could represent precision medicine for more severe phenotypes caused by *KCNQ2* and *KCNQ3* LoF variants. Further work is required to identify additional patients carrying *KCNQ3* or *KCNQ2* pathogenic LoF variants retaining responsiveness to BHB *in vitro* and who may benefit from KD treatment *in vivo*.

## Conclusion

In conclusion, we describe the first missense LoF pathogenic variant located in the S_4_ segment of *KCNQ3* as a cause of BFNE. In contrast to BFNE-*KCNQ3* variants previously described, functional and modeling data suggest that the M240R variant primarily affects the voltage sensitivity of heteromeric channels. Our study provides a pharmacological rationale for investigating the use of KD in patients with DEE caused by *KCNQ3* and *KCNQ2* LoF variants.

## Data Availability Statement

The raw data supporting the conclusions of this article will be made available by the authors, without undue reservation.

## Ethics Statement

The studies involving human participants were reviewed and approved by the Columbia University Irving Medical Center. Written informed consent to participate in this study was provided by the participants’ legal guardian/next of kin. Written informed consent was obtained from the individual(s), and minor(s)’ legal guardian/next of kin, for the publication of any potentially identifiable images or data included in this article.

## Author Contributions

FM, MT, and TS conceived the study, analyzed the data, and wrote the manuscript. EC, MC, and DG analyzed the data and wrote the manuscript. FM, LC, VB, MS, EH, AM, NL, and LB performed the research and analyzed the data. All authors contributed to the article and approved the submitted version.

## Conflict of Interest

The authors declare that the research was conducted in the absence of any commercial or financial relationships that could be construed as a potential conflict of interest.

## Footnotes

1 http://spdbv.vital-it.ch/

2 http://www.pymol.org/

## References

[B1] Allen A. S. Berkovic S. F. Cossette P. Delanty N. Dlugos D. Eichler E. E. (2013). De novo mutations in epileptic encephalopathies. *Nature* 501 217–221. 10.1038/nature12439 23934111 PMC3773011

[B2] Allen N. M. Mannion M. Conroy J. Lynch S. A. Shahwan A. Lynch B. (2014). The variable phenotypes of KCNQ-related epilepsy. *Epilepsia* 55 e99–e105. 10.1111/epi.12715 25052858

[B3] Ambrosino P. Freri E. Castellotti B. Soldovieri M. V. Mosca I. Manocchio L. (2018). Kv7.3 Compound Heterozygous Variants in Early Onset Encephalopathy Reveal Additive Contribution of C-Terminal Residues to PIP2-Dependent K(+) Channel Gating. *Mol. Neurobiol.* 55 7009–7024. 10.1007/s12035-018-0883-5 29383681

[B4] Bassi M. T. Balottin U. Panzeri C. Piccinelli P. Castaldo P. Barrese V. (2005). Functional analysis of novel KCNQ2 and KCNQ3 gene variants found in a large pedigree with benign familial neonatal convulsions (BFNC). *Neurogenetics* 6 185–193. 10.1007/s10048-005-0012-2 16235065

[B5] Bosch D. G. Boonstra F. N. de Leeuw N. Pfundt R. Nillesen W. M. de Ligt J. (2016). Novel genetic causes for cerebral visual impairment. *Eur. J. Hum. Genet.* 24 660–665. 10.1038/ejhg.2015.186 26350515 PMC4930090

[B6] Brown D. A. Adams P. R. (1980). Muscarinic suppression of a novel voltage-sensitive K+ current in a vertebrate neurone. *Nature* 283 673–676. 10.1038/283673a0 6965523

[B7] Charlier C. Singh N. A. Ryan S. G. Lewis T. B. Reus B. E. Leach R. J. (1998). A pore mutation in a novel KQT-like potassium channel gene in an idiopathic epilepsy family. *Nat. Genet.* 18 53–55. 10.1038/ng0198-53 9425900

[B8] Deciphering Developmental Disorders (2017). Prevalence and architecture of de novo mutations in developmental disorders. *Nature* 542 433–438. 10.1038/nature21062 28135719 PMC6016744

[B9] Fister P. Soltirovska-Salamon A. Debeljak M. Paro-Panjan D. (2013). Benign familial neonatal convulsions caused by mutation in KCNQ3, exon 6: a European case. *Eur. J. Paediatr. Neurol.* 17 308–310. 10.1016/j.ejpn.2012.10.007 23146207

[B10] Fusco C. Frattini D. Bassi M. T. (2015). A novel KCNQ3 gene mutation in a child with infantile convulsions and partial epilepsy with centrotemporal spikes. *Eur. J. Paediatr. Neurol.* 19 102–103. 10.1016/j.ejpn.2014.08.006 25278462

[B11] Grinton B. E. Heron S. E. Pelekanos J. T. Zuberi S. M. Kivity S. Afawi Z. (2015). Familial neonatal seizures in 36 families: clinical and genetic features correlate with outcome. *Epilepsia* 56 1071–1080. 10.1111/epi.13020 25982755

[B12] Grozeva D. Carss K. Spasic-Boskovic O. Tejada M. I. Gecz J. Shaw M. (2015). Targeted Next-Generation Sequencing Analysis of 1,000 Individuals with Intellectual Disability. *Hum. Mutat.* 36 1197–1204. 10.1002/humu.22901 26350204 PMC4833192

[B13] Hirose S. Zenri F. Akiyoshi H. Fukuma G. Iwata H. Inoue T. (2000). A novel mutation of KCNQ3 (c.925T–>C) in a Japanese family with benign familial neonatal convulsions. *Ann. Neurol.* 47 822–826. 10.1002/1531-8249(200006)47:6<822::aid-ana19>3.0.co;2-x 10852552

[B14] Hou P. Kang P. W. Kongmeneck A. D. Yang N. D. Liu Y. Shi J. (2020). Two-stage electro-mechanical coupling of a KV channel in voltage-dependent activation. *Nat. Commun.* 11:676. 10.1038/s41467-020-14406-w 32015334 PMC6997178

[B15] Jensen M. O. Jogini V. Borhani D. W. Leffler A. E. Dror R. O. Shaw D. E. (2012). Mechanism of voltage gating in potassium channels. *Science* 336 229–233. 10.1126/science.1216533 22499946

[B16] Jentsch T. J. (2000). Neuronal KCNQ potassium channels: physiology and role in disease. *Nat. Rev. Neurosci.* 1 21–30. 10.1038/35036198 11252765

[B17] Kanaumi T. Takashima S. Iwasaki H. Itoh M. Mitsudome A. Hirose S. (2008). Developmental changes in KCNQ2 and KCNQ3 expression in human brain: possible contribution to the age-dependent etiology of benign familial neonatal convulsions. *Brain Dev.* 30 362–369. 10.1016/j.braindev.2007.11.003 18166285

[B18] Kim K. S. Duignan K. M. Hawryluk J. M. Soh H. Tzingounis A. V. (2016). The voltage activation of cortical KCNQ channels depends on global PIP2 levels. *Biophys. J.* 110 1089–1098. 10.1016/j.bpj.2016.01.006 26958886 PMC4788708

[B19] Knupp K. G. Wirrell E. C. (2018). Treatment strategies for dravet syndrome. *CNS Drugs* 32 335–350. 10.1007/s40263-018-0511-y 29594870

[B20] Ko A. Jung D. E. Kim S. H. Kang H. C. Lee J. S. Lee S. T. (2018). The Efficacy of Ketogenic Diet for Specific Genetic Mutation in Developmental and Epileptic Encephalopathy. *Front. Neurol.* 9:530. 10.3389/fneur.2018.00530 30061856 PMC6054992

[B21] Kothur K. Holman K. Farnsworth E. Ho G. Lorentzos M. Troedson C. (2018). Diagnostic yield of targeted massively parallel sequencing in children with epileptic encephalopathy. *Seizure* 59 132–140. 10.1016/j.seizure.2018.05.005 29852413

[B22] Lauritano A. Moutton S. Longobardi E. Tran Mau-Them F. Laudati G. Nappi P. (2019). A novel homozygous KCNQ3 loss-of-function variant causes non-syndromic intellectual disability and neonatal-onset pharmacodependent epilepsy. *Epilepsia Open* 4 464–475. 10.1002/epi4.12353 31440727 PMC6698674

[B23] Li H. Li N. Shen L. Jiang H. Yang Q. Song Y. (2008). A novel mutation of KCNQ3 gene in a Chinese family with benign familial neonatal convulsions. *Epilepsy Res.* 79 1–5. 10.1016/j.eplepsyres.2007.12.005 18249525

[B24] Long S. B. Tao X. Campbell E. B. MacKinnon R. (2007). Atomic structure of a voltage-dependent K+ channel in a lipid membrane-like environment. *Nature* 450 376–382. 10.1038/nature06265 18004376

[B25] Lutas A. Yellen G. (2013). The ketogenic diet: metabolic influences on brain excitability and epilepsy. *Trends Neurosci.* 36 32–40. 10.1016/j.tins.2012.11.005 23228828 PMC3534786

[B26] Maljevic S. Vejzovic S. Bernhard M. K. Bertsche A. Weise S. Docker M. (2016). Novel KCNQ3 mutation in a large family with benign familial neonatal epilepsy: a rare cause of neonatal seizures. *Mol. Syndromol.* 7 189–196. 10.1159/000447461 27781029 PMC5073621

[B27] Manville R. W. Papanikolaou M. Abbott G. W. (2018). Direct neurotransmitter activation of voltage-gated potassium channels. *Nat. Commun.* 9:1847. 10.1038/s41467-018-04266-w 29748663 PMC5945843

[B28] Manville R. W. Papanikolaou M. Abbott G. W. (2020). M-Channel activation contributes to the anticonvulsant action of the ketone body beta-Hydroxybutyrate. *J. Pharmacol. Exp. Ther.* 372 148–156. 10.1124/jpet.119.263350 31757819 PMC6994816

[B29] Miceli F. Soldovieri M. V. Ambrosino P. Barrese V. Migliore M. Cilio M. R. (2013). Genotype-phenotype correlations in neonatal epilepsies caused by mutations in the voltage sensor of K(v)7.2 potassium channel subunits. *Proc. Natl. Acad. Sci. U.S.A.* 110 4386–4391. 10.1073/pnas.1216867110 23440208 PMC3600471

[B30] Miceli F. Soldovieri M. V. Joshi N. Weckhuysen S. Cooper E. Taglialatela M. (2018). “KCNQ2-Related Disorders,” in *GeneReviews((R))*, eds Adam M. P. Ardinger H. H. Pagon R. A. Wallace S. E. Bean L. J. H. Stephens K. (Seattle, WA: University of Washington). 20437616

[B31] Miceli F. Soldovieri M. V. Joshi N. Weckhuysen S. Cooper E. C. Taglialatela M. (2017). “KCNQ3-Related Disorders,” in *GeneReviews((R))*, eds Adam M. P. Ardinger H. H. Pagon R. A. Wallace S. E. Bean L. J. H. Stephens K. (Seattle, WA: University of Washington). 24851285

[B32] Miceli F. Soldovieri M. V. Lugli L. Bellini G. Ambrosino P. Migliore M. (2009). Neutralization of a unique, negatively-charged residue in the voltage sensor of K V 7.2 subunits in a sporadic case of benign familial neonatal seizures. *Neurobiol. Dis.* 34 501–510. 10.1016/j.nbd.2009.03.009 19344764

[B33] Miceli F. Striano P. Soldovieri M. V. Fontana A. Nardello R. Robbiano A. (2015). A novel KCNQ3 mutation in familial epilepsy with focal seizures and intellectual disability. *Epilepsia* 56 e15–e20. 10.1111/epi.12887 25524373

[B34] Millichap J. J. Park K. L. Tsuchida T. Ben-Zeev B. Carmant L. Flamini R. (2016). KCNQ2 encephalopathy: features, mutational hot spots, and ezogabine treatment of 11 patients. *Neurol Genet.* 2:e96. 10.1212/NXG.0000000000000096 27602407 PMC4995058

[B35] Neubauer B. A. Waldegger S. Heinzinger J. Hahn A. Kurlemann G. Fiedler B. (2008). KCNQ2 and KCNQ3 mutations contribute to different idiopathic epilepsy syndromes. *Neurology* 71 177–183. 10.1212/01.wnl.0000317090.92185.ec 18625963

[B36] Pisano T. Numis A. L. Heavin S. B. Weckhuysen S. Angriman M. Suls A. (2015). Early and effective treatment of KCNQ2 encephalopathy. *Epilepsia* 56 685–691. 10.1111/epi.12984 25880994

[B37] Rauch A. Wieczorek D. Graf E. Wieland T. Endele S. Schwarzmayr T. (2012). Range of genetic mutations associated with severe non-syndromic sporadic intellectual disability: an exome sequencing study. *Lancet* 380 1674–1682. 10.1016/S0140-6736(12)61480-9 23020937

[B38] Sands T. T. Balestri M. Bellini G. Mulkey S. B. Danhaive O. Bakken E. H. (2016). Rapid and safe response to low-dose carbamazepine in neonatal epilepsy. *Epilepsia* 57 2019–2030. 10.1111/epi.13596 27888506

[B39] Sands T. T. Miceli F. Lesca G. Beck A. E. Sadleir L. G. Arrington D. K. (2019). Autism and developmental disability caused by KCNQ3 gain-of-function variants. *Ann. Neurol.* 86 181–192. 10.1002/ana.25522 31177578

[B40] Schroeder B. C. Kubisch C. Stein V. Jentsch T. J. (1998). Moderate loss of function of cyclic-AMP-modulated KCNQ2/KCNQ3 K+ channels causes epilepsy. *Nature* 396 687–690. 10.1038/25367 9872318

[B41] Singh N. A. Westenskow P. Charlier C. Pappas C. Leslie J. Dillon J. (2003). KCNQ2 and KCNQ3 potassium channel genes in benign familial neonatal convulsions: expansion of the functional and mutation spectrum. *Brain* 126 (Pt 12), 2726–2737. 10.1093/brain/awg286 14534157

[B42] Soh H. Pant R. LoTurco J. J. Tzingounis A. V. (2014). Conditional deletions of epilepsy-associated KCNQ2 and KCNQ3 channels from cerebral cortex cause differential effects on neuronal excitability. *J. Neurosci.* 34 5311–5321. 10.1523/JNEUROSCI.3919-13.2014 24719109 PMC3983807

[B43] Soldovieri M. V. Boutry-Kryza N. Milh M. Doummar D. Heron B. Bourel E. (2014). Novel KCNQ2 and KCNQ3 mutations in a large cohort of families with benign neonatal epilepsy: first evidence for an altered channel regulation by syntaxin-1A. *Hum. Mutat.* 35 356–367. 10.1002/humu.22500 24375629

[B44] Soldovieri M. V. Castaldo P. Iodice L. Miceli F. Barrese V. Bellini G. (2006). Decreased subunit stability as a novel mechanism for potassium current impairment by a KCNQ2 C terminus mutation causing benign familial neonatal convulsions. *J. Biol. Chem.* 281 418–428. 10.1074/jbc.m510980200 16260777

[B45] Sun J. MacKinnon R. (2020). Structural basis of human KCNQ1 modulation and gating. *Cell* 180 340-347.e9. 10.1016/j.cell.2019.12.003 31883792 PMC7083075

[B46] Symonds J. D. Zuberi S. M. Stewart K. McLellan A. O’Regan M. MacLeod S. (2019). Incidence and phenotypes of childhood-onset genetic epilepsies: a prospective population-based national cohort. *Brain* 142 2303–2318. 10.1093/brain/awz195 31302675 PMC6658850

[B47] Tinel N. Lauritzen I. Chouabe C. Lazdunski M. Borsotto M. (1998). The KCNQ2 potassium channel: splice variants, functional and developmental expression. Brain localization and comparison with KCNQ3. *FEBS Lett.* 438 171–176. 10.1016/s0014-5793(98)01296-4 9827540

[B48] Tzingounis A. V. Nicoll R. A. (2008). Contribution of KCNQ2 and KCNQ3 to the medium and slow afterhyperpolarization currents. *Proc. Natl. Acad. Sci. U.S.A.* 105 19974–19979. 10.1073/pnas.0810535105 19060215 PMC2604953

[B49] Uehara A. Nakamura Y. Shioya T. Hirose S. Yasukochi M. Uehara K. (2008). Altered KCNQ3 potassium channel function caused by the W309R pore-helix mutation found in human epilepsy. *J. Membr. Biol.* 222 55–63. 10.1007/s00232-008-9097-5 18425618

[B50] Wang H. S. Pan Z. Shi W. Brown B. S. Wymore R. S. Cohen I. S. (1998). KCNQ2 and KCNQ3 potassium channel subunits: molecular correlates of the M-channel. *Science* 282 1890–1893. 10.1126/science.282.5395.1890 9836639

[B51] Weckhuysen S. Mandelstam S. Suls A. Audenaert D. Deconinck T. Claes L. R. (2012). KCNQ2 encephalopathy: emerging phenotype of a neonatal epileptic encephalopathy. *Ann. Neurol.* 71 15–25. 10.1002/ana.22644 22275249

[B52] Zara F. Specchio N. Striano P. Robbiano A. Gennaro E. Paravidino R. (2013). Genetic testing in benign familial epilepsies of the first year of life: clinical and diagnostic significance. *Epilepsia* 54 425–436. 10.1111/epi.12089 23360469

